# Good Laboratory Practices: Tyl Responds

**DOI:** 10.1289/ehp.0901495R

**Published:** 2010-02

**Authors:** Rochelle W. Tyl

**Affiliations:** RTI International Research Triangle Park, North Carolina, E-mail: rwt@rti.org

I am responding to the comments by vom Saal and Myers on my commentary (Tyl 2009), which was in response to their commentary (Myers et al. 2009). Our dietary BPA mouse study was performed under Organisation for Economic Co-operation and Development (OECD) Test Guideline 416 (OECD 2001) and Good Laboratory Practices (GLPs) (OECD 1998), with in-house quality control, and formal RTI quality assurance, and expert Developmental & Reproductive Toxicology (DART) panel oversight. We submitted all of our summary data online with our published mouse study (Tyl et al. 2008). I also discussed our BPA data, especially prostate weights (ventral and dorsolateral lobes separately) and ages of animals at scheduled necropsy, two areas of concern to vom Saal and Myers, in my commentary (Tyl 2009). We found no BPA effects on mouse prostate weights at any dietary dose, from 3 μg/kg/day to 600 mg/kg/day, whereas vom Saal and colleagues reported increased prostate weights at 2 and 20 μg/kg/day BPA, administered on gestational days 11–17 (Nagel et al. 1997). Vom Saal and Myers suggested that prostate weights were very large in our control mice (Tyl et al 2008), in his view, likely due to prostatitis and/or poor dissection techniques resulting in extraneous tissue left on the prostates. We provided histopathologic confirmation of low (normal) rates of prostatitis, no increased incidences or severities from BPA, and no evidence of extraneous tissue from examination of the prostate paraffin block faces and histology slides. For animal ages at termination, we initially presented approximate ages of our animals at demise because we were not aware of their concerns at that time. In a letter to the editor of *Toxicological Sciences*, where the multigenerational BPA rat and mouse studies (Tyl et al. 2002, 2008) were published, I (Tyl 2009) explained in great detail the ages of our F_0_, F_1_, and F_2_ animals at scheduled necropsy; the ages of the F_1_ animals varied at most by 3 weeks in all groups, based on when the F_0_ animals mated during the 2-week mating period, the need to have all F_1_ offspring exposed for at least 8 weeks during the prebreeding period, and the need for all F_1_s to be available for pairing in all groups at the same time. FDA auditors recently spent 11 days at RTI (30 March to 9 April 2009) inspecting our BPA rat (Tyl et al. 2002) and mouse (Tyl et al. 2008) multigenerational reproductive toxicity study data and records, with no study findings.

It is clear that vom Saal and Myers apparently still do not understand or appreciate the discipline, power, importance, and usefulness of GLPs on study design, performance, documentation, and interpretation (which is why GLP-compliant studies are preferentially used in formal hazard identification and risk assessment).

The effects of dose levels, route, timing (life stages), and duration of BPA exposures on reported early and late effects, and whether there is a linkage between the low-dose early end points from short-term, small, basic exploratory studies and the outcomes from long-term guideline studies, need to be evaluated. Long-term, robust oral studies (Ashby et al. 1999; Cagen et al. 1999) and guideline-compliant oral multigenerational studies (Ema et al. 2001; Tyl et al. 2002, 2008), regardless of sponsorship, have not confirmed the low-dose effects reported in the basic studies, nor any long-term consequences anticipated from these reported early effects. Determination of whether there is hazard or risk of BPA to humans and wildlife from low, environmentally relevant doses by relevant exposure routes is based on available appropriate data. To date, governmental and other organizational hazard, risk, and weight-of-evidence assessments have concluded, based on the data, that there is no evidence of any adverse effects from oral BPA at low doses.
